# The comparison of the process of manual and robotic positioning of the electrode performing radiofrequency ablation under the control of a surgical navigation system

**DOI:** 10.1038/s41598-020-64472-9

**Published:** 2020-05-25

**Authors:** A. A. Levin, D. D. Klimov, A. A. Nechunaev, A. A. Vorotnikov, L. S. Prokhorenko, E. V. Grigorieva, D. A. Astakhov, Y. V. Poduraev, D. N. Panchenkov

**Affiliations:** 1 Moscow State University of Technology “STANKIN”, 1 Vadkovsky per., Moscow, 127055 Russian Federation; 2 Moscow State University of Medicine and Dentistry named after A.I. Evdokimov, 20/1 Delegatskaya ul., Moscow, 127473 Russian Federation

**Keywords:** Cancer imaging, Biomedical engineering

## Abstract

This study is aimed at the comparison of the process of manual and robotic positioning of the electrode performing radiofrequency ablation under the control of a surgical navigation system. The main hypothesis of this experiment was that the use of a collaborative manipulator (KUKA iiwa) will allow to position the active part of the electrode relative to the center of the tumor more accurately and from the first attempt. We also monitor the stability of the electrode′s velocity during insertion and consider some advantages in ergonomics using the robotic manipulator. We use three more criteria to compare the surgeon's and robotic performance, unlike other studies, where only the target point's accuracy criterion is observed. The main idea is to examine the movement parameters of the electrode that can lead to potential patient trauma. Sphere-shaped tumor phantoms measuring 8 mm in diameter were filled with contrast and inserted in bovine livers. 10 livers were used for the robotic experiment and an equal quantity for manual surgery. The livers were encased in silicone phantoms designed to imitate the liver position in a real patient's abdominal cavity. Analysis of CT data gave the opportunity to find the entry and the target point for each tumor phantom. This data was loaded into a surgical navigation system that was used to track and record the position of the RF-electrode during the operation for further analysis. The standard deviation of points from the programmed linear trajectory totaled in the average 0.3 mm for the robotic experiment and 2.33 mm for the manual operation with a maximum deviation of 0.55 mm and 7.99 mm respectively. Standard deviation from the target point was 2.69 mm for the collaborative method and 2.49 mm for the manual method. The average velocity was 2.97 mm/s for the manipulator and 3.12 mm/s for the manual method, but the standard deviation of the velocity relative to the value of the average velocity was 0.66 mm/s and 3.05 mm/s respectively. Thus, in two criteria out of three, the manipulator is superior to the surgeon, and equality is established in one. Surgeons also noticed advantages in ergonomics performing the procedure using the manipulator. This experiment was produced as part of the work on the developing of a robotic multifunctional surgical complex. We can confirm the potential advantages of using collaborative robotic manipulators for minimally invasive surgery in case of practice for cancer treatment.

## Introduction

Minimally invasive surgery is becoming a standard in the treatment of cancer of internal organs^1–6^. The main advantages are considered to be short hospitalization periods and a reduction in the postoperative recovery time. Modern studies also show that there are groups of patients at particular risk (diabetic, with an increased body mass index (BMI > 35) for whom minimally invasive techniques are optimal. Also, statistics show that the proportion of such patients is steadily increasing^7^. This indicates, first of all, that extensive development of methods of minimally invasive surgery is coming.

Minimally invasive technologies have found their primary application in the treatment of primary and secondary tumors of the liver. Treatment of focal lesions of the liver is especially important when you consider that up to 1.4 million (2012) of new cases of colorectal cancer are registered in the world every year^8^, which occupies the 2nd place in the structure of oncological diseases, while 20% of initially diagnosed patients have stage IV with liver metastases. This cancer is the third most common among men and the second among women^9^. Another 50% of patients will have metastases, most to the liver, in the future. 80% of primary and 60% of secondary liver cancer are not resectable. During radical surgery on the liver, the incidence of postoperative complications is 19–43%, and postoperative mortality is about 10%^10^.

The technique of radiofrequency ablation is widely used in the treatment of cancer of internal organs, in particular - the liver. High efficiency is produced by a large selection of electrode shapes and modes of exposure. It retains all the advantages of minimally invasive surgery techniques. However, despite the positive aspects that it brings to the patient, doctors have to face certain difficulties.

The main one is the precise positioning of the active electrode of the ablator. The surgeon under the control of ultrasound should get the electrode to the target point, that is, to combine the center of the working area of the electrode with the center of the tumor. The complexity of this process is aggravated by the fact that it is extremely desirable to do this at the first attempt, as precisely as possible, without damaging the vascular secretion elements of the liver. In some cases, the operation can take a long time (from 20 to 40 minutes), which requires special efforts from the surgeon to maintain the immobility of the ablator electrode.

An obvious suggestion could be an attempt to replace the surgeon with a robotic manipulator. Its main advantages can be noted: more accurate positioning and orientation of the electrode relative to the intended path of motion, reduction of the amplitude of oscillations in the process of movement, fixation in the desired position during operation, removing the electrode exactly through the input channel to minimize additional damage. Recently, robot-assisted surgery is gaining popularity, more and more surgeons use it to treat colorectal cancer^11^. The study of S. Janki proved that the question of ergonomics for operating personnel is of great importance^12^. The problem for the surgeon is to maintain the posture of immobility for a long time, and often uncomfortable positioning of the hands, to minimize the shaking of the instrument^13^. Unlike freehand manual insertion, robotic insertion offers the advantage of stable needle posture at a given angle during needle targeting and insertion. Another advantage is that surgeons may remain seated and unexposed to radiation during the procedure^14^. That should be a more ergonomic procedure for the physicians in case of prolonged operations.

For a more accurate spatial determination of the target point, navigation using radial diagnostic methods with high spatial resolution is necessary. The best method for assessing the state of the liver is intravenous contrast-enhanced computed tomography. The method allows to simultaneously visualize the vascular anatomy and focal liver changes with high accuracy. Separately, it is worth noting that the use of computed tomography in the 3D mode allows to determine the entry point and the target point in such a way as to avoid finding the vessels in the path of motion. In addition to the key points, it is possible to define a safety zone, which the electrode should not leave during the operation. The use of the manipulator will allow controlling the position of the electrode relative to this zone, thus increasing the safety of the intervention.

The purpose of the experiment described below was to compare the manual and the robotic performance of positioning the RFA electrode using four different criteria:

1. The standard deviation of points from a given linear path is the amount of deviation at each point from its projection onto the center line (model), using the least squares method.
  Shows the deviation from the trajectory during positioning and is useful to check that the electrode moves along the planned trajectory not damaging the soft tissue.
2. The standard deviation of points from a given target point at the time of ablation.
  Shows the ability to reach the planned target point inside the tumor.
3. The standard deviation of points from the midpoint at the time of ablation.
  Used to monitor the jitter of the electrode during the ablation process.
4. The standard deviation of the average velocity - the magnitude of the deviation from the average movement velocity.
  Shows the stability of the electrode movement along the planned trajectories.

Currently, some manipulators allow to solve the problem partly. For example, Perfint MAXIO. The Perfint MAXIO is an image-guided, physician controlled stereotactic accessory to a Computed Tomography (CT) system. Perfint MAXIO is intended for the stereotactic spatial positioning and orientation of an end effector and instrument guide to assist in manual advancement of one or more instruments such as rigid straight needles and probes during CT guided percutaneous procedures on organs and anatomical structures in the thorax, abdomen and pelvis^15^. This system is widely used; however, it has its drawbacks. In particular, it does not use a robotic input of an electrode into an internal organ, leaving this stage of the operation to the surgeon and performing only the navigational function^16^. This technology may be useful for clinical CT-guided biopsy and RFA, when accuracy may have an impact on the outcome. It makes achievable the improved accuracy of complex needle insertions. Also, a robotic interventional radiologist assistance platform can improve target ablation coverage^17^.

## Methods

This study was approved by Interuniversity Ethics Committee (Russia, Moscow, http://ethicmke.ru/). Authors confirm that all methods were performed in accordance with the relevant guidelines and regulations: "Use of experimental animals, and human participants" by Springer Nature Scientific Reports. The following equipment was used for the experiment: a FOTEK electrosurgical high-frequency AB-150 apparatus, a 7.5 mm needle electrode, an optical navigation system “Multitrack”, a KUKA iiwa 14 robotic manipulator, a Aquilion 64 (Canon) computed tomography scanner.

### FOTEK AB-150

The device is designed for volumetric monopolar and bipolar radiofrequency coagulation (ablation) of biological tissue with an automatic stop, as well as for ligation (brewing) of large blood vessels using controlled radiofrequency coagulation of tissues with their simultaneous mechanical compression by a clamp. The device in the modes of monopolar and bipolar radiofrequency ablation allows volume coagulation of tissues located deep from the surface of the organ, under automatic control of the effectiveness of the devitalization of the tumor formation. The volume and geometry of the coagulant are governed by the size and shape of the working part of the instrument (electrode), the selected power and time of exposure. The device heats the soft tissues adjacent to the electrode with a high-frequency current of a particular form under the control of tissue impedance. High-frequency current can be supplied once or cyclically for a specified time. With a single supply, the current heats the tissues adjacent to the electrode to a temperature of 95–110 °С and at the moment of complete drying of the tissues the flow of current automatically stops. When cycling the machine for a set amount of time, it repeats the following cycles: supply of high-frequency current before heating and full drying of tissues, holding the pause for moistening (reducing impedance) and resuming the amount of high-frequency current. The cyclic process of applying current, heating and drying, repeated many times, allows to maintain a high temperature in the ablation zone for a long time and reach a large amount of coagulated tissue^18^.

### MULTITRACK

The stereotaxic surgical navigation system “MULTITRACK” in the version “MULTITRACK-T”. The error in determining the coordinates of the centers of the reflecting spheres - passive markers, is not more than 0.7 mm. The distance at which the registration of a surgical instrument with a given accuracy is ensured is the range of 1.5–2.0 m. The dimensions of the illuminated area (the area of the operative field) at a distance of 1.5 m from the block of the optical recording system are at least 1.0 m3. The Multitrack navigation system is designed to perform interventions in neurosurgery and traumatology and provides control and visualization of the movement of surgical instruments in the surgical field relative to the target of the organ being operated in real time using 3D CT/MRT images obtained during preliminary examination of the patient^19^.

**CT-Scanner Aquilion 64 (Canon)** was used to obtain a series of images for subsequent registration with the navigation system. The scan was carried out with the capture of the entire volume of the studied organ with a slice thickness of 1 mm, followed by the reconstruction of 0.5 mm images, with the construction of 2D and 3D reconstructions for more accurate core registration with the navigation system. To improve the imaging volume and formation of blood vessels, the X-ray contrast agent Omnipaque 370 was applied.

### KUKA iiwa

Robotic arm with a load capacity of 14 kg and a radius of action of 820 mm. The robot has seven controlled axes with position repeatability of ±0.15 mm.

### Experiment

**Figure 1:**
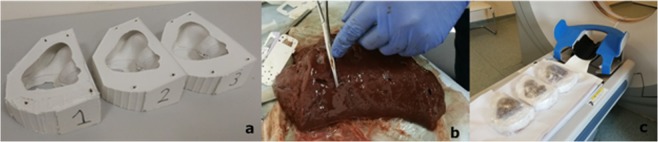
(**a**) The phantom silicone mold, (**b**) Liver preparation, (**c**) The phantom CT scan process.

At this stage of the experiment, we used bovine liver with implanted foreign bodies, which are radiologically different from the liver parenchyma to create a biological liver phantom with a tumor. To do this, we first used the fruits of olives, beans and foam balls. We placed these structures intraparenchymal by incision of the liver capsule and pushing them 20, 30, and 50 mm deep into the parenchyma. At the same time, we tried to position the channels horizontally to avoid displacement of implants along the channel when the phantom was moved. (Fig. 1b) During the first experiments, we found that some of these tumor phantoms are not suitable for these purposes due to minimal densitometric difference with the liver parenchyma. For a more accurate visualization, we began to use only foam balls, soaked with a solution of X-ray contrast agent Omnipaque. The choice of foam rubber is explained by the ease with which it is inserted into the parenchyma, practically by the absence of its migration through the channel due to its porous structure and rough surface, and the possibility to inject the contrast agent into it.

For better visualization of the vascular structures of the liver, the contrast agent was injected into the arteries of the beef liver fragment. The arteries were sutured to prevent its leakage and the formation of false structures that mimic blood vessels. Thus, it was possible to simulate the arterial and venous phases of the contrasting of the vessels of the liver and the tumor phantom. A fragment of the liver, with sutured arteries, filled with the contrast agent and implanted foam balls, was placed in a silicone mold, repeating the shape of the liver. Silicone molds with a cavity were specially manufactured so that the inside cavities would replicate the shape of a human liver.

The mold had two windows. The upper window with the size of 7 * 8 cm was used for laying the liver and its orientation in the mold cavity. The bottom window with dimensions of 12 * 14 cm served to ensure the contact of the liver with the conventional passive electrode of the apparatus for radiofrequency ablation (Fig. 1a). To eliminate displacement, a fragment of bovine liver was selected that would fit or be slightly larger in size than the cavity in the silicone mold. Next, the mold with the liver was tightly wrapped with polyethylene to prevent displacement or loss of the liver during transport between the CT device and the operating table. Thus, ten phantoms were prepared for each of the two methods - manual and robotic, each of which contained two foam balls. Next, a CT scan was performed of several phantoms placed parallel to the table (Fig. 1c). The 3D-model of the obtained samples was created.

The data obtained from the CT were recorded and analyzed in the Multitrack navigation system software. The analysis allowed to determine the target points (centers of phantom neoplasms) and entry points to the phantoms. These two points for each phantom neoplasm determined the trajectory of the manipulator. The deviation of the working tool was measured with respect to a straight line passing through the two key points.

We conducted a series of experimental operations of radiofrequency ablation on a biological phantom of the liver with a tumor. All operations were divided into 2 groups. In the first group, the positioning of the instrument for the RFA in the liver parenchyma with a tumor phantom according to the data obtained by CT of the liver phantom was performed by a robotic manipulator. In the second group, the positioning of the instrument was performed directly by the surgeon manually, which requires a significant amount of training, hand-eye coordination, 2D to 3D extrapolation skills^20^.

At the first stage, a 3D-model of bovine liver with a phantom of the tumor (foam ball) was built on the basis of the CT data in the Multitrack software. At the same time, the location of the phantom of the tumor in the parenchyma, its connection with the vascular structures, the distance from the edges and the surface of the liver were evaluated.

A silicone mold with a biological liver phantom wrapped in polyethylene was placed on the operating table. The bottom window of the silicone mold was released from the plastic lash by cutting it along the contour of the hole. After that, the mold was placed on the passive electrode connected to the apparatus of radiofrequency ablation. (Fig. 3a)

**Figure 2:**
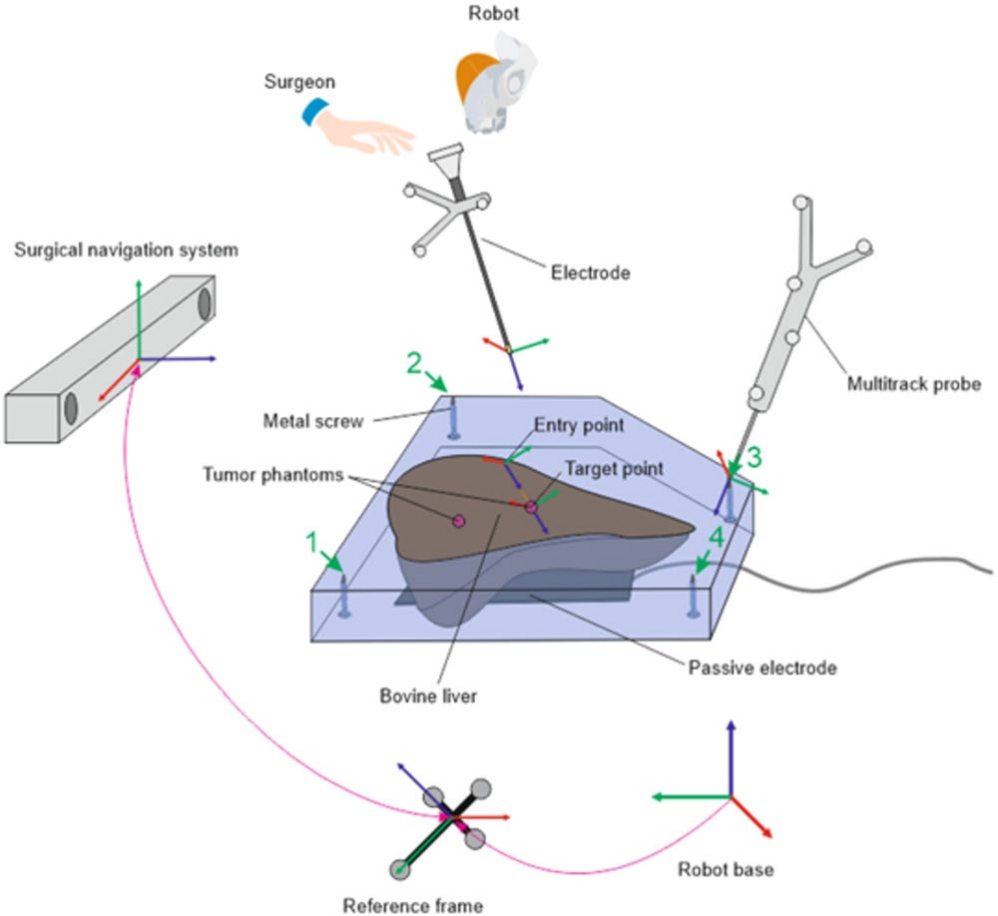
Schematic diagram of the preparation for the experiment.

Determining the coordinates of the phantom and its subsequent binding to the model obtained using CT, was carried out using the tools of the Multitrack navigation system, providing an error of 0.7 mm. To register the phantom in the workspace of the surgical navigation system, the following procedure was performed. Each phantom was equipped with four metal screws arranged so that their tips extend from the top of the silicone mold. They are clearly visible on the phantom model obtained based on CT and are convenient to use in point-based registration. A specialized probe equipped with IR reflectors of the Multitrack system was subsequently brought up to the tips of the screws for the conventional point-based registration procedure (Fig. 2). The origin of the surgical navigation system is located in the center of the reference frame. The reference frame is firmly fixed to the table on which the phantom is located (Fig. 2).

After the phantom was registered in the workspace of the surgical navigation system the coordinate transformation from the navigation equipment to the robot was determined. In order to achieve this, the instrument with an electrode equipped with IR reflectors was fixed on the robot flange. The first step was to calibrate the instrument so that the robot’s tool center point (TCP) was at the tip of the electrode. This procedure was carried out by regular means of the iiwa robot controller. Then the instrument was registered in the Multitrack navigation system by rotating the instrument around the new TCP in various planes using the robot. It is a built-in function of the Multitrack software, which records the position of the frame with IR reflectors during movement, after which it determines the common center of rotation. Based on this data, the relative position of IR reflectors and TCP was established. The next step is to determine the coordinate transformation from the Multitrack system to the robot. The robot sequentially positioned the tool at several points with a known relative position, and the navigation system recorded these points in its coordinate system. Based on the collected data, the coordinate transformation from the Multitrack system to the robot base was found using the singular value decomposition method^21^.

After these procedures, it became possible to transfer key points from the navigation system to the robot controller. Using the navigation system, the coordinates of the two key points were determined - the target and the entry point to the phantom of the internal organ. The surgeon solved this problem based on medical images obtained on CT, through the graphical interface of the Multitrack software. To compute the position of the target in robot space it is necessary to compute first the transformation between the CT image space and the robot space – this procedure is called the registration transformation. It is based on the alignment procedure using the key points. These obtained coordinates were entered into the control program of the robotic system, which determined the trajectory of the robot’s movement.

**Figure 3:**
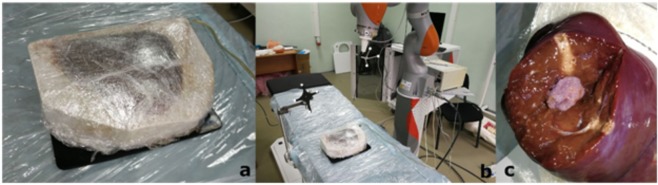
(**a**) Emplacement of liver, (**b**) Performing an operation, (**c**) Result of ablation.

The first group of experiments was carried out using a robotic manipulator. The control program guided the active part of the electrode mounted on the flange of the manipulator to the target point. Upon completion of the procedure, the robot was given a command to remove the electrode. The movement was carried out along the same trajectory while conducting the track ablation - heating the electrode input-output channel, to reduce blood loss. Then the radiofrequency ablation device was turned on manually (Fig. 3b).

The second group of experiments had a similar setup. The surgeon was provided with a graphical interface that is included in the software of the Multitrack system, used for brain biopsy. During the manual movement of the electrode, the deviation from the trajectory, orientation, and target point were graphically displayed on the screen. It allowed the surgeon to adjust his movements in real-time.

Choosing a position and making sure that the instrument is held in the correct position, the surgeon began his insertion into the liver parenchyma. The depth of insertion of the instrument and its location in the liver parenchyma was estimated according to the data displayed on the monitor. The location of the instrument was estimated by aligning the instrument axis with the target axes on the monitor screen, and the depth of its insertion by numbers showing the distance to a previously determined target point located behind the phantom of the tumor so that the instrument passes through the center of the phantom of the tumor and somewhat stands behind it. After guiding the instrument to a predetermined point, the surgeon performed the ablation procedure. Then the surgeon took the instrument out of the parenchyma while also conducting the track ablation. One of the results of the ablation procedure is shown in the photo below (Fig. 3c).

In both groups of experiments, the positions of the electrode were recorded with the surgical navigation system with a frequency of ~100 Hz.

Liver preparations were transferred to the morphological study after performing experimental operations. The purpose was to compare the histological picture of the distribution of coagulation necrosis after RFA when positioning electrodes by a robot and a surgeon, and to assess the accuracy of the electrode entering the tumor model.

## Results

The data obtained through the surgical navigation system were transformed for further analysis in the MATLAB software. The analysis was carried out according to the criteria described above^22^.

The first criterion revealed the following results: the standard deviation of points from a given linear trajectory was 2.33 mm for a manual experiment and 0.3 mm for a robotic experiment. Thus, it is obvious that the robot maintains a linear trajectory more stably than a surgeon^23^ - the robot has surpassed the surgeon by more than 7 times. The gathered data revealed a 7.99 mm maximum deviation of for the surgeon against 0.55 mm for the robotic experiment, which is more than 14 times smaller. This difference is due to the fact that even for an experienced surgeon it is very difficult to maintain a straight-line trajectory of the input-output of the electrode, avoiding sharp displacements. Thus, among 20 trajectories performed by the surgeon, in four, displacements exceeding 7 mm were recorded. This allows to confirm the original proposal and conclude that in practice the robotized method is less dangerous for the patient and allows to avoid unnecessary injury^24^.

The standard deviation of points from a given target point at the time of ablation is also a critical parameter. It allows to assess how accurately the electrode is positioned relative to the target point — usually the center of the neoplasm. Proper positioning allows to avoid necrosis of healthy tissues of the body, causes less damage to the body while preserving its functionality. The indicators for this criterion were 2.49 mm in the manual experiment and 2.69 mm in the robotized one. This is the first criterion where the capabilities of a surgeon and a manipulator turned out to be comparable. In this case it must be taken into account that the performance of the robot can be improved since the error is not due to the physical limitations of the manipulator, but to various sources of errors, in particular, the procedure of the coordinate transformation between the navigation system and the robot.

Deviations from the midpoint at the time of the ablation were also calculated. This indicator describes how stably a person and the robot hold the electrode during the procedure. The unstable position of the electrode causes additional damage. The surgeon showed a 1.08 mm standard deviation of points from the midpoint at the time of ablation. As for the robot, this parameter does not make sense, since the ablation procedure was carried out in a stationary position with the brakes on at the joints of the manipulator. Thus, the data obtained by the navigation system does not reflect the actual displacements of the electrode but corresponds to the error of the Multitrack system. Here comes another problem – moving – the target might move because of tissue deformation, displacement or respiratory motion. Currently there are two ways of minimizing the effects of moving. First is instructing the patient to hold his breath during the insertion^20^. Second, in case of general anesthesia, physicians may start the ablation procedure right after CT is completed, while the patient is still lying on the CT table. It also should be suggested that in the case of a long-term operation by a person, an additional increase in this indicator is possible, due to fatigue and nervous tension. For a robot, this indicator remains constant regardless of the time of the operation.

The last indicator tested was the standard deviation from the average velocity - the deviation from the average velocity of the electrode's movement. An assumption was made in advance that, like the deviation from the midpoint, the velocity deviation for the robot will be constant, regardless of external factors - for example, differences in the densities of the internal organ. Experiments have shown that in this respect, the robot is two or more times better than a human^25^. The average velocity of the electrode input was 3.12 mm/s and 2.97 mm/s for the robot at a given target velocity of 3 mm/s. However, the standard deviation from the average velocity in a manual experiment was 3.05 mm/s, and this is comparable with the given velocity. The robot was able to maintain the desired velocity 4–5 times more precisely - its standard deviation was 0.66 mm/s.

The mathematical approach to the analysis of the results showed that in three of the four indicators the robot predictably surpassed the person, relative equality was achieved in one indicator. For convenience, the results were tabulated (Table 1). The successful application of robots in such operations is linked to the quality of their preliminary calibration^26–29^.

**Table 1:** Results of the manual and the robotic experiments.

	Deviation from the trajectory, mm		Deviation from the target point, mm	Deviation from the midpoint, mm	Deviation from average velocity, mm/s	
	σ	MAX	σ	σ	AVG	σ
Manual	2.33	7.99	**2.49**	1.08	3.12	3.05
Robotic	**0.3**	**0.55**	2.69	—	**2.97**	**0.66**

Takao Hiraki, MD reports that his experiment with Zerobot Robotic System revealed similar accuracies at one criterion - deviation from the target point (mean accuracy, 1.6 and 1.4 mm, respectively)^14^. A. Patriciu also reports of achievement of 1.7 mm of mean accuracy with standard deviation of 0.8 mm in his “Randomized Patient Study of Robotic-Assisted RF Ablation of Liver Tumors”^20^.

As an additional confirmation of the possibility of using the robot for radiofrequency ablation, liver samples were transferred to the histology laboratory for additional analysis and comparison.

**Figure 4:**
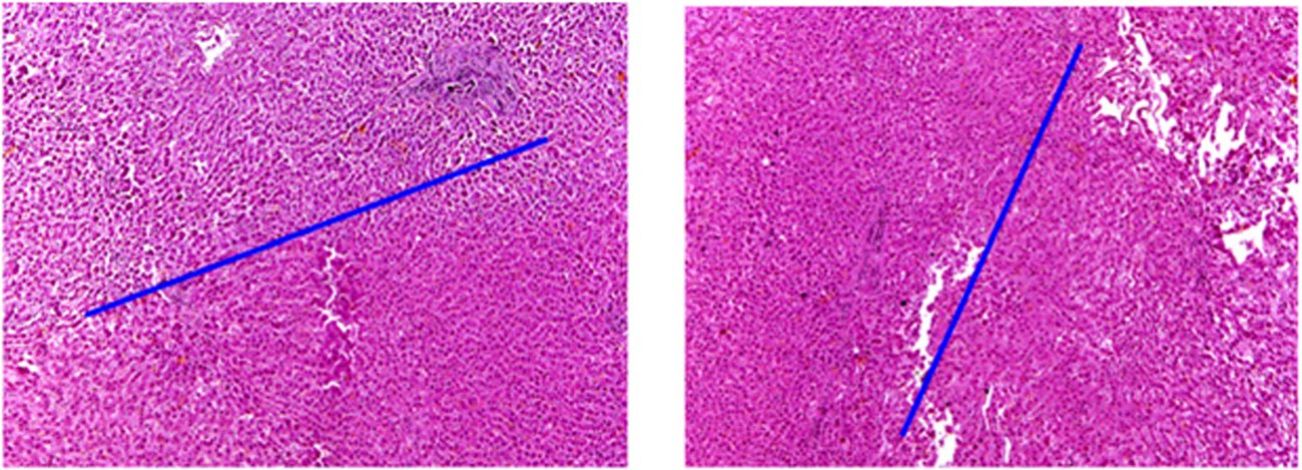
(**a**) Sections of the liver in a manual experiment, (**b**) Sections of the liver in the robotic experiment, stained with hematoxylin and eosin, magnification 60×.

The results of the study of the manual experiment (Fig. 4a). show the border of the intact (bottom) and necrotic (top) liver tissue (indicated by the blue line). Below - the lobular and beam structure of the liver is preserved, hepatocytes, sinusoids, portal tracts are not changed. Above - coagulative necrosis of all structures of the liver tissue. The lobular and beam structure of the liver is impaired, the hepatocytes in a state of necrosis (hyperchromic, with plasmorexis, karyopicnosis and karyorexis) form cellular detritus, the structures of the portal tract (the connective tissue of the vascular wall, bile ducts) are necrotic and destroyed. There was also no difference in the coagulation necrosis in the wound channel when performing track ablation after the procedure.

The results of the study of the robotic experiment are shown in Fig. 4b, the boundary of the intact (left) and necrotized (right) liver tissue is indicated by the blue line. On the left - the lobular and beam structure of the liver is preserved, hepatocytes, sinusoids, portal tracts are not changed. On the right predominantly coagulative necrosis of all the structures of the liver tissue is shown. The lobular and beam structure of the liver is impaired, the hepatocytes in a state of necrosis (hyperchromic, with plasmorexis, karyopicnosis and karyorexis) form cellular detritus, the structures of the portal tract (the connective tissue of the vascular wall, bile ducts) are necrotized and destroyed.

Thus, histological examination revealed no discrepancies between the amount of coagulation necrosis in the area of the phantom of the tumor when a robot and a surgeon perform RFA. This suggests that for the ablation procedure itself, the robotic approach affects the tissues in the same way as the surgeon, replacing the navigation and transport functions exclusively. However, as experiments have shown, the robot can perform this function with higher rates.

Also, the operating personnel noted the ease of use of the manipulator, the reduction of psychological and physical stress after the procedures with its participation compared with manual procedures. Thus, in terms of ergonomics, the robotic method has surpassed the manual one.

## Conclusions

This experiment was produced as part of the work on the “Digital Robotic Framework”. In a joint project MSMSU named after A.I. Evdokimov and MSTU STANKIN are implementing a multifunctional automated robotic system (MARS), in particular, intended for use in the field of neurosurgery and abdominal surgery, and the Articulated Arm Braking Mechatronic Machine, the concept of which was shown in^30^. It is built on the basis of a manipulator that moves medical instruments to the required position and orientation relative to the operated site.

This research shows the advantages of performing the RF-ablation procedure using the robotic-assisted method. Deviation from the target point criterion demonstrates equality between the robot and the surgeon. Other criteria reveal the robot to be five times better in case of velocity stability and 14 times more accurate in case of deviation from the trajectory. So the robotic manipulator potentially would harm the patient less and reduce the postoperative recovery time.

The manipulator did not yield to the surgeon by any of the stated criteria, for most of them it turned out to be much more effective than experienced oncologists. The team of authors will continue further work on the framework of the project described above.
